# Drug use and driving behaviors among drivers with and without alcohol-related infractions

**DOI:** 10.1590/2237-6089-2019-0034

**Published:** 2020-10-08

**Authors:** Juliana N. Scherer, Jaqueline B. Schuch, Marcelo R. Rocha, Vanessa Assunção, Roberta B. Silvestrin, Vinícius S. Roglio, Renata P. Limberger, Tanara R. V. Sousa, Flavio Pechansky

**Affiliations:** 1 Centro de Pesquisa em Álcool e Drogas Hospital de Clínicas de Porto Alegre Universidade Federal do Rio Grande do Sul Porto AlegreRS Brazil Centro de Pesquisa em Álcool e Drogas, Hospital de Clínicas de Porto Alegre, Universidade Federal do Rio Grande do Sul (UFRGS), Porto Alegre, RS, Brazil.; 2 Programa de Pós-Graduação em Psiquiatria e Ciências do Comportamento UFRGS Porto AlegreRS Brazil Programa de Pós-Graduação em Psiquiatria e Ciências do Comportamento, UFRGS, Porto Alegre, RS, Brazil.; 3 Programa de Pós-Graduação em Ciências Farmacêuticas UFRGS Porto AlegreRS Brazil Programa de Pós-Graduação em Ciências Farmacêuticas, UFRGS, Porto Alegre, RS, Brazil.

**Keywords:** Driving under the influence, traffic accidents, law enforcement, alcohol, psychoactive substances

## Abstract

**Introduction:**

Brazil is one of the countries with the highest rates of alcohol-related traffic infractions, but little is known about the profile of the drivers who commit them. Identifying the characteristics of impaired drivers is essential for planning preventive actions.

**Objective:**

To compare drug use and driving behavior profiles of drivers with and without alcohol-related infractions.

**Methods:**

178 drivers stopped at routine roadblocks were assessed by traffic agents who conducted standard roadblock procedures (document verification; request of a breathalyzer test [BT]). Drug use and driving behavior data were collected through semi-structured interviews. Subjects were divided into three groups: drivers who refused the BT (RDs, n = 72), drivers who tested positive on the BT (PDs, n = 34), and drivers who had committed other infractions (ODs, n = 72).

**Results:**

The proportion of alcohol use in the last year was higher among RDs (100%) than in the PD and OD groups (97.1% and 72.2% respectively, p < 0.001). Lifetime prevalence of cannabis and cocaine use for the overall sample was 44.3% and 18.2%, respectively. Fewer individuals in the OD group (31.5%) reported having been stopped at roadblocks in the previous year compared to the PDs (55.9%) and RDs (48.6%, p = 0.03). However, a higher proportion of RDs reported drunk driving in the same period (87.5%; PD 69.7%; OD 26.9%; p < 0.001).

**Conclusion:**

Essential differences among groups were observed. RDs had a higher proportion of alcohol use and drunk driving in the previous year; drivers who fit into this particular group may be unresponsive or less responsive to social deterrence and enforcement actions.

## Introduction

Traffic collisions (TCs) associated with drunk driving are still a cause of concern worldwide, especially in low and middle-income countries (LMIC), where TCs account for a great proportion of the increase in disability-adjusted life years (DALYs).^1^ Among the strategies developed to reduce drunk driving, inspection barriers with random breath testing are shown to be a cost-effective approach, reducing alcohol-related TCs by about 20%.^2^ According to the 2009 Global Status Report on Road Safety, 49% of countries that were analyzed have specific laws in place that prohibit drinking and driving with a blood alcohol concentration (BAC) equal to or higher than 0.05g/dl,^3^ which is considered by the World Health Organization (WHO) as the best practice for traffic enforcement.^4^

In Brazil, laws and policies related to drunk driving have changed significantly over the years. Although the first national law concerning alcohol consumption by drivers was established in 1941, it was only in 1997 that a BAC limit of 0.06g/dl was established.^5 , 6^ In 2008, Brazil implemented stricter laws, with a BAC threshold of zero.^7^ Enforcement of the zero tolerance policy prioritizes use of roadside breath tests as the standard procedure for detecting drunk driving; however, when breath testing is not available or cannot be performed, traffic agents may also rely on other types of evidence, such as witness accounts and visible signs and symptoms of impairment.^8^ In theory, recognition of signs and symptoms of alcohol impairment by traffic agents can be used as the sole evidence for conviction of a traffic crime or offense, but in practice this procedure is almost never used, due to lack of training both by police officers and members of the court. When drivers are asked to perform a breath test during a roadblock, a number of different outcomes are possible, as shown in Table 1 . Legal loopholes exist that allow drivers to refuse to take the breath test, claiming the right not to self-incriminate. Drivers who refuse are subject to the same fines and administrative penalties as drivers with a breath alcohol concentration (BrAC) between 0.04 and 0.35 mg/L of breath.^9 - 11^

**Table 1:** - Breath testing outcomes and consequences according to Brazilian legislation

Breathalyzer outcome	Sanction*
Driver accepts the breath testing	
BrAC < 0.04mg/L	None
BrAC from 0.04 to 0.34mg/L	Infraction (art. 165) + fine (R$2934.70) + driver’s license suspended for up to 12 months
BrAC ≥ 0.35mg/L	Infraction (art. 165) + traffic crime (art. 306) + fine (R$2934.70) + driver’s license suspended for up to 3 years
Driver refuses to take the breath test	Infraction (art. 165) + fine (R$2934.70) + driver’s license suspended for up to 12 months
Drivers with signs and symptoms recognized by traffic agents, irrespective of breath testing outcome	Infraction (art. 165) + traffic crime (art. 306) + fine (R$2934.70) + driver’s license suspended for up to 3 years

BrAC = breath alcohol concentration.

* Considering drivers who have not committed DUI offenses in the previous year. Drivers who reoffend within a 1-year period are subject to more severe penalties.

At the time of data collection, US$ 1.00 = R$ 3.20.

Evidence shows that it is possible to deter alcohol-impaired drivers, mainly through police enforcement and severe punishments.^2 , 12 , 13^ However, even with a “dry law” in place and an increase in traffic enforcement in Brazil, the number of alcohol-related collisions increased by over 1,000 between 2007 and 2015. In this regard, both Brazilian and international literature suggests that traffic law enforcement together with strict sanctions are effective for reducing the rates of drunk driving traffic offenses.^14 - 16^ One recent paper evaluated how different countries deal with a refusal situation and concluded that countries with lower levels of drunk driving deaths seem to have achieved a level of societal agreement that driving under the influence (DUI) is deviant, generating social stigma against DUI that allows legislation to be enforced.^10^ Moreover, In the United States, for example, it is against the law to refuse a breathalyzer test. All states have an implied-consent law, whereby operating a motor vehicle obligates the motorist to comply with field sobriety tests. Driving is thus considered a privilege, not a right. In Brazil, the legal possibility of refusal can be perceived as a less severe penalty, and therefore be less effective for preventing reoffending and for achieving social deterrence.

Besides having more permissive policies regarding traffic safety when compared with developed countries, LMIC also have less scientific data regarding the characteristics and specific of offenders.^10 , 17 , 18^ This points to a need for further enforcement of drinking and driving laws and to the urgency of assessing and reviewing the target audience. In this respect, it is important to study the characteristics of drivers who refuse to take breath tests in order to propose better enforcement policies targeting this specific group. Our hypothesis is that drivers who refuse to take the breathalyzer test during roadblocks are a risk group for drunk-driving. In this sense, the possibility of refusing the test associated with the mild penalties for refusing the test could make drivers prone to repeated risky behaviors. Therefore, we aimed to compare the differences in drug use profile and driving behaviors between drivers who refused to perform the breathalyzer test and drivers with other types of infractions.

## Methods

### Ethical considerations

This study was approved by the Institutional Review Board at the Hospital de Clínicas de Porto Alegre (HCPA/GPPG N.14-0685, CAAE 39604114.3.0000.5327). All participants provided written consent. Agreeing or refusing to participate in the study did not add/subtract any legal measures or administrative penalties. All screening results and data collected during the interview were used exclusively for study purposes.

### Study design and sampling

This was a cross-sectional roadside survey, and data were collected during checks carried out at inspection barriers by traffic agents from the Department of Transportation of the state of Rio Grande do Sul (DETRAN-RS) and the Federal Highway Police (PRF-RS) in the metropolitan area of Porto Alegre, Brazil, as part of standard operating procedures. The days and places of data collection were chosen by convenience, based on police availability and their own planning. The study was conducted between April and September 2016, from Tuesday to Saturday nights (11 PM to 5 AM).

All drivers stopped at routine roadblocks went through a standard protocol consisting of a document check and breath testing. During data collection, a total of 3,321 drivers were stopped and subjected to the initial procedures at checkpoints. Breath tests were conducted using Alco-Sensor IV (Intoximeters Inc., St. Louis, USA) or BAF-300 (Elec Inc., São Paulo, Brazil) models. In addition to following their standard procedures, traffic agents were trained and instructed to assess drivers for possible signs and symptoms of intoxication using a standardized evaluation protocol. This protocol covers 10 dimensions of intoxication symptoms related to changes to psychomotor skills (orientation, mental status, coordination, gestures/signs, breath smell, appearance of the eyes and face, speech, attitude, body reaction). The protocol was adapted from the DRUID project and from previous Brazilian protocols.^19^

Inclusion criteria were: committing any of the infractions that would prevent drivers from returning to the road and continue driving, namely: a) having a BrAC over 0.04 mg/L of breath; b) refusing to take the breath test; c) presenting signs and symptoms of intoxication by alcohol and/or other psychoactive substances (PAS) (as assessed by the standard protocol); d) not having a valid driver’s license; e) any other legal criterion that would prevent them from driving (such as driving without their driver’s license, not having paid car taxes, burnt out bulb or a poor connection, including headlights, brake lights, or turn signals). The use of very strict and conservative inclusion criteria was for ethical reasons, since it would not be appropriate to let a driver in an impaired condition or who self-reported being under the influence return to the road.

In order to compare different driver profiles, the subjects were divided into three groups according to type of traffic infraction: RD, or drivers who refused to perform the breath test; PD, or drivers with a positive BrAC (BrAC > 0.04 mg/L); and OD, or drivers who committed other types of infraction not involving impaired conditions for driving (e.g., not having a valid driver’s license; not having a car license).

### Procedures

Drivers who met inclusion criteria were invited to participate by a senior investigator. Drivers received a brief explanation of the study and data on apparent age and traffic infraction were collected for all these individuals. If a driver consented to participation, he or she was taken by a trained interviewer to a private place dedicated to data collection. All consenting drivers signed informed consent forms. The interview comprised a semi-structured questionnaire with questions about age, sex, education, income, driving experience, vehicle type, and history of DUI. After completing the questionnaire, which took about 20 minutes, drivers were referred to another private space where drug screening tests were conducted (data not shown in the present paper). The questionnaire and forms were filled out in an electronic format using tablets running the Open Data Kit (ODK), a tool for development and storage of survey forms supported by ongoing research at the University of Washington’s Department of Computer Science & Engineering. The data collected were sent to the common server. All identifying data were encrypted.

After participating in the research, drivers were directed to the responsible traffic agent in order to complete the routine roadblock operation.

### Statistical analysis

Categorical variables are expressed as absolute and relative frequencies, and proportions and associations were analyzed using the chi-squared test. We investigated the normality of distribution of continuous data using a histogram and the Shapiro-Wilk test. Continuous variables were expressed as median and interquartile range and were compared using the Kruskal-Wallis test followed by Dunn’s post-hoc test. Statistical analyses were conducted using SPSS, v.18 (IBM Corp., Armonk, NY).

## Results

### Sampling

Of the 3,321 drivers stopped at the checkpoints, 309 met the inclusion criteria and 179 (57.9%) agreed to participate in the study. There were no differences in apparent age or sex between study participants and those who refused to participate; however, breathalyzer results were associated with participant agreement, the proportion of zero BrAC (< 0.04mg/L) was higher among those who agreed to participate (30.7% vs. 19.2%) while the proportion of test refusal was lower (36.9% vs. 56.9%; p < 0.001) in this group ( Table 2 ). One participant dropped out of the study during the interview and was therefore excluded. The final sample consisted of 178 drivers. Of these, 34 (19.1%) had a positive BrAC result; 72 (40.4%) refused to take the breath test; and 72 (40.4%) had committed other types of infractions.

**Table 2:** - Association between apparent sociodemographic characteristics and agreement to participate in the study among drivers who fulfilled the inclusion criteria (n = 309)

		Participation agreement		
			
Variable	Total (n = 309)	Yes (n = 179)	No (n = 130)	p-value
Apparent age				
18 to 29 years	97 (33.9)	69 (38.5)	28 (26.2)	0.068
30 to 59 years	178 (62.2)	105 (58.7)	73 (68.2)	
60 years or above	11 (3.8)	5 (2.8)	6 (5.6)	
Sex (male)	245 (85.7)	159 (88.8)	86 (80.4)	0.072
Breathalyzer result (mg/L of breath)				
Zero (< 0.04)	80 (25.9)	55 (30.7)*	25 (19.2)*	< 0.001
Administrative infraction (0.05 to 0.34)	31 (10)	25 (14)	6 (4.6)	
Crime (> 0.34)	5 (1.6)	5 (2.8)	0 (0)	
Refused testing	140 (45.3)	66 (36.9)*	74 (56.9)*	
Not filled out by traffic agent^†^	53 (17.2)	28 (15.6)	25 (19.2)	-

Data expressed as absolute and relative frequencies, n (%), and compared using the chi-square test.

* Adjusted residual > |2|.

^†^ This field was not filled out in 34.3% of the forms. For drivers who agreed to participate in the study, these data were obtained during the interview.

### Demographic characteristics and driving profile

Drivers were mostly male (89.3%), with a median age of 33 years. Most drove a car or pickup/SUV (83.1%) daily (65.2%), had spent less than 12 years in education (70.2%), and reported an average income equivalent to 4.1 Brazilian minimum wages per month (minimum wage in 2016: R$ 880, equivalent to US$ 275 that year). There were a few differences in demographic characteristics between the three groups of drivers ( Table 3 ). For instance, a lower proportion of drivers with other types of infractions (excluding those related to impaired driving) had a valid driver’s license; nonetheless, the type of vehicle and weekly frequency of driving were similar to the other groups ( Table 3 ).

**Table 3:** Demographic characteristics and driving profiles of drivers with different infractions (n = 178)

Variable	Positive breathalyzer (n = 34)	Refused breathalyzer (n = 72)	Other reasons (n = 72)	p-value
Sex, male*	32 (94.1)	62 (86.1)	65 (89.3)	0.434
Age^†^	36 [28.0-46.5]	32 [26.2-42.7]	32.5 [25.2-39.7]	0.274
Education, 12+ years*	9 (26.5)	24 (33.3)	20 (27.8)	0.687
Monthly income (R$)^†‡^	2500 [1500-4500]	3000 [1500-5000]	2200 [1600-3500]	0.332
Vehicle*				
Car/pickup	24 (70.6)	65 (90.3)	59 (81.9)	
Motorcycle or similar	6 (17.6)	4 (5.6)	8 (11.1)	0.162
Truck or bus	4 (11.8)	3 (4.2)	12 (6.7)	
Driver’s license*	34 (100.0)	67 (93.1)	59 (81.9)^§^	0.008
Drove daily*	21 (61.8)	54 (75)	41 (59.9)	0.068

* Data expressed as absolute and relative frequencies, n (%), and compared using the chi-squared test.

^†^ Data expressed as median [interquartile range] and compared using the Kruskal-Wallis test.

^‡^ At the time of data collection, US$ 1.00 = R$ 3.20.

^§^ Adjusted residual > |2|.

### Self-reported drug use and signs and symptoms of intoxication

Out of the total sample, 88.2% and 68.8% reported having consumed alcohol in the last year and in the previous 24h, respectively. Drivers who refused the breathalyzer test had a higher prevalence of alcohol use in the previous year compared with the overall sample prevalence (p < 0.001). Among the subjects who refused the breath test, fewer reported having had alcohol in the previous 24h (93.1%) than among the BrAC positive group (97%). Only 17.3% of drivers with other types of infractions reported alcohol use in the previous 24h ( Table 4 ).

**Table 4:** - Self-report of drug use and signs and symptoms of intoxication among drivers with different infractions (n = 178)

Variable	Positive breathalyzer (n = 34)	Refused breathalyzer (n = 72)	Other reasons (n = 72)	p-value
Alcohol				
Last year	33 (97.1)	72 (100.0)*	52 (72.2)*	< 0.001
Last 3 months^†^	33 (97.1)	72 (100.0)*	52 (72.2)*	< 0.001
Last 24h (n = 157)^‡^	32 (97.0)*	67 (93.1)*	9 (17.3)*	< 0.001
Cannabis				
Lifetime	15 (44.1)	31 (43.1)	31 (43.1)	0.994
Last 3 months^†^	4 (26.7)	20 (64.5)*	10 (32.3)	0.012
Last 24h (n = 34)^‡^	2 (50.0)	9 (45.0)	4 (40.0)	0.936
Cocaine				
Lifetime	5 (14.7)	11 (15.3)	16 (22.2)	0.477
Last 3 months^†^	0	4 (36.4)	2 (12.5)	0.149
Last 24h (n = 6)^‡^	0	1 (25.0)	1 (50.0)	0.540
Amphetamines				
Lifetime	2 (5.9)	2 (2.8)	4 (5.6)	0.658
Last 3 months^†^	0	1 (50.0)	2 (50.0)	0.449
Last 24h (n = 3)^‡^	0	0	0	-
Total number of signs and symptoms^§^	2 [0-4]	2 [1-5]	0 [0-1]	0.192

Data expressed as absolute and relative frequencies, n (%), and compared using the chi-squared test, unless otherwise specified.

* Adjusted residual > |2|.

^†^ The total n for the analysis of use during the last 3 months is the subset of subjects who reported using the drug during their lifetimes.

^‡^ The total n for the analysis of use during the last 24 hours is the subset of subjects who reported using the drug during the previous 3 months.

^§^ Data expressed as median [interquartile range] and compared using the Kruskal-Wallis test.

The most prevalent illicit drugs used in the previous year were cannabis (44.3%) and cocaine (18.2%). Of the 77 participants who reported having used cannabis in the previous year, 34 (44.1%) and 15 (19.5%) also reported cannabis use in the previous three months and in the last 24 hours, respectively. Of the 32 participants who reported having used cocaine in the previous year, six (18.7%) and two (6.2%) also reported cocaine use in the previous three months and in the last 24 hours, respectively. Except for alcohol consumption and cannabis use in the previous three months, there were no differences in the other variables related to drug use between the groups of drivers ( Table 4 ).

The total number of signs and symptoms of intoxication assessed by traffic agents was similar between groups (p = 0.192), with drivers presenting a median of one sign or symptom in total ( Table 4 ).

### Impaired driving behaviors and beliefs

Among the drivers who reported alcohol consumption in the previous year, 63.7% (n = 100) drove shortly after alcohol consumption at least once during this period. Drivers who refused the breathalyzer test presented a higher proportion of this behavior (p < 0.001). Twenty-five subjects reported driving under the influence of drugs in the previous year, accounting for 27.8% of the whole sample that reported drug use in the previous year (n = 90), with no differences between groups ( Table 5 ). Over 50% of the overall sample also reported having been the passenger of a driver impaired by alcohol and/or drugs in the last year.

**Table 5:** Impaired driving behaviors and beliefs among drivers with different infractions (n = 178)

Variable	Positive breathalyzer (n = 34)	Refused breathalyzer (n = 72)	Other reasons (n = 72)	p-value
Drunk driving (last year) (n = 157)^†^	23 (69.7)	63 (87.5)*	14 (26.9)*	< 0.001
Drugged driving (last year) (n = 90)^†^	4 (23.5)	13 (36.1)	8 (21.6)	0.350
Ride with an impaired driver (last year)	14 (41.2)	37 (51.4)	40 (55.6)	0.384
Stopped at a roadblock (last year)	19 (55.9)	35 (48.6)	23 (31.9)*	0.033
Believes enforcement can reduce drunk driving	26 (76.5)	48 (66.7)	45 (62.5)	0.361
Believes enforcement can reduce drug driving	27 (79.4)	52 (72.2)	50 (69.4)	0.562

Data expressed as absolute and relative frequencies, n (%), and compared using the chi-squared test.

* Adjusted residual > |2|.

^†^ The total n for these analyses is the subset of subjects who reported using the drug in the previous year.

With regard to enforcement variables, fewer drivers with other types of infraction (31.5%) reported having been stopped at a roadblock during the previous year compared to the other two groups (55.9% of those with positive BrAC; 48.6% of those who refused the breathalyzer; p = 0.03). Enforcement was considered by 66.9% and 72.3% of the overall sample as an effective measure for reducing drunk and drugged driving, respectively, with no difference between groups ( Table 5 ).

## Discussion

In the present study, we compared drug use profiles, behaviors, and beliefs related to impaired driving among drivers with and without alcohol-related infractions stopped at Brazilian roadblocks, focusing on the characteristics of drivers who refused to take the breathalyzer test. We found that those who refused the test had a higher proportion of alcohol use and drunk driving behavior in the previous year, even though most drivers in this group had already been stopped before at roadblocks and believed in the effectiveness of enforcement.

Several studies have reported a high prevalence and severity of PAS use and dependence among drivers with a history of driving under the influence.^20 - 23^ Current data in Brazil estimate that around 50% of the population aged 18 years or more has used alcohol in the previous year,^24^ compared to 88.2% in our sample. This discrepancy was even higher in the RD group, where the proportion of individuals reporting alcohol use was almost two times greater than the national prevalence. Although we found no difference in drug use between groups, the overall sample reported a higher proportion of cannabis and cocaine use than those reported for the general population in previous reports (2.4% and 2%, respectively).^24^ Since all drug use data were self-reported, it is possible that they were underestimated. One hypothesis for this result relies on the fact that the sample was approached during night roadblocks, as evidence suggest that the prevalence of alcohol and drug positive drivers increase on week nights and weekends.^25^ Besides, the prevalence of alcohol and drug use in Rio Grande do Sul is higher than in most of the country’s states. However, history of drug use is not the sole risk for DUI; indeed, comprehensive assessment of drug use profiles, cognitive traits, and personality characteristics seems to be the best way to identify citizens who are higher-risk drivers, as well as to assess those who are not responsive to traditional enforcement measures.^20 , 26^

Policies and enforcement strategies against impaired driving vary greatly between countries and are generally drafted based on local needs. In this context, understanding the profile of drivers with a history of drunk or drugged driving is essential to designing specific approaches to this public problem, since the mere importation of policies or programs from other countries would not cater to our current needs in Brazil. Notwithstanding, there are few studies worldwide evaluating the profile of impaired drivers in LMIC. International studies from Europe and the United States that assessed offending drivers showed a high proportion of men,^27 - 30^ and of drivers of cars rather than other models.^30^ Mean age varied slightly between studies but remained within a range of 30-50 years.^29 , 30^ Some exceptions were also observed with younger^27^ and older subjects.^31^ Although average income may not be directly assessed through studies, there is a high proportion of employed or self-employed individuals.^29 , 30^ In previous Brazilian and LMIC studies, drivers with traffic infractions also present a similar sociodemographic profile, with most identifying as young male adults from middle or upper social classes.^21 , 32 , 33^ In this sense, the sociodemographic profile of our sample is in line with international and previous Brazilian data, but the fact that we evaluated a sample from southern Brazil must be considered. However, even with a similar sociodemographic profile of traffic offenders, drivers from LMIC and developed counties have different perspectives on traffic safety.

In our results, we noted a significant difference between groups regarding driving behavior and beliefs. The high proportion of drunk and drugged driving among drivers who refused the breath test suggests a potential risk among this population. Moreover, the RD group also had a lower proportion of drivers who believe that traffic enforcement can reduce drunk driving. Previous data show that drivers with low perceived risk of legal consequences commit more alcohol-impaired driving offenses.^34 , 35^ Moreover, drivers with higher perceived risk are more compliant with enforcement and treatment countermeasures and are more likely to report safer driving behaviors.^35^ Altogether, the higher risk profile of refusing drivers seen in our results, the mild sanctions, and the weak enforcement system in Brazil may be favoring the continuity of risk behaviors. Therefore, our results support the position that driving laws should penalize refusal as a traffic crime in order to achieve reduction of repeat offenses and to improve social deterrence.

Recently, Pechansky et al. showed how the differences in enforcement and sanctions for DUI could be compared to road traffic mortality rates and to social and economic development parameters in different countries.^10^ According to these authors, developed countries with strict laws and enforcement actions have higher social deterrence regarding DUI and, consequently, lower traffic mortality rates. In this sense, several authors have discussed the influence of social deterrence on DUI behavior. While there is still no consensus, most studies suggest that social deterrence is an effective way to reduce negative outcomes in traffic.^36 , 37^ Some studies have shown that repeat offenders in Brazil are usually aware of the traffic laws and risks of TCs involving specific behaviors, which supports the fact that there is lack of social deterrence among Brazilian drivers.^38 , 39^ In our study, 43.3% of drivers with alcohol-related infractions reported they had been stopped at a roadblock in the previous year. Although most said they believed in the effectiveness of enforcement, they were being fined for drinking and driving at the time of data collection. Likewise, 69.7% and 87.5% of PD and RD who reported alcohol consumption in the previous year were recidivist DUI offenders, respectively. When combined, these results indicate that the usual traffic enforcement actions may have no effect on this group of subjects.

Although considered an administrative offense, drivers who refuse a breath test and do not exhibit additional evidence of intoxication can see this possibility as a way to circumvent legislation. It is worth noting that all groups were similarly assessed for signs and symptoms of impairment. The literature is conflicting with regard to the validity of signs and symptoms as the sole evidence for being under the influence.^40 - 44^ In our study, drivers with positive BrAC and drivers who refused the breath test had the same number of signs and symptoms as drivers with negative BrAC, which suggests that, in its present form, the assessment performed by traffic agents was not accurate for identifying DUI among drivers. Although some subjects reported being under the influence of PAS other than alcohol, this proportion was not different between groups. It is therefore important to reevaluate the policies regarding recognition of signs and symptoms in drivers and to invest in the specific training of agents to detect them – perhaps training drug recognition experts, as an example of the strategies used by some developed countries.^45 , 46^

One of the limitations of this study is the fact that it was conducted exclusively with drivers who were not be allowed to return to the road and continue driving, which prevents comparisons with a control group. Another limitation is the fact that data were collected on the roadside, where time and infrastructure for data collection is limited. This study therefore lacks information regarding the personality and cognitive aspects of drivers, which would be important to better define the risky profiles among groups. However, even with a cross-sectional design and a small sample size, we were able to detect initial evidence that distinguishes between drivers committing different infractions, suggesting that drivers who refuse breath tests could be a high-risk group.

## Conclusion

This study showed that drivers committing different types of infractions have different profiles of drug use and different behaviors and beliefs related to traffic risk. These results are in line with other studies suggesting that drivers with different risk behaviors are very heterogeneous. In this sense, the authors highlight the need for robust longitudinal studies with LMIC drivers with a history of DUI to create combined measures that could lead to early identification and prevention of negative traffic outcomes, including DUI recidivism and traffic accidents. Lack of social deterrence and strict sanctions, together with the possibility of refusing breath testing may be important factors that influence DUI behavior in Brazil, including higher risk drivers.
